# Socioeconomic status and alcohol use disorders across the lifespan: A co-relative control study

**DOI:** 10.1371/journal.pone.0224127

**Published:** 2019-10-17

**Authors:** Susanna Calling, Henrik Ohlsson, Jan Sundquist, Kristina Sundquist, Kenneth S. Kendler

**Affiliations:** 1 Center for Primary Health Care Research, Skåne University Hospital, Lund University, Malmö, Sweden; 2 Department of Clinical Sciences Malmö, Lund University, Malmö, Sweden; 3 Virginia Institute for Psychiatric and Behavioral Genetics, Virginia Commonwealth University, Richmond, Virginia, United States of America; 4 Department of Psychiatry, Virginia Commonwealth University, Richmond, Virginia, United States of America; University of Jyvaskyla, FINLAND

## Abstract

**Objectives:**

Alcohol use disorders (AUD) is well known to aggregate in families and is associated with socioeconomic status (SES). The objective was to study the effect of education, income and neighborhood SES in adulthood on AUD, and to explore whether the potential associations were confounded by shared familial factors, by using a co-relative control design.

**Methods:**

Data on AUD was drawn from the Swedish inpatient and outpatient care registers; prescription drug register; and crime data. Through national population registers we collected information on income, education and neighborhood SES at age 25, 30, 35 and 40 years in all individuals born in Sweden between 1950 and 1980. Each sex-specific stratum consisted of approximately 750,000–1,200,000 individuals, who were followed for AUD for a mean follow-up time ranging between 10 and 15 years until the end of 2013. Cox proportional hazards models were used to investigate the risk of AUD as a function of income, education and neighborhood SES in the general population and in pairs of first cousins and full siblings within the same sex, who differed in their exposure to the SES measure.

**Results:**

Higher educational level, higher income and higher neighborhood SES were all associated with a reduced risk for AUD for both males and females in all ages. The potentially protective effect remained but was attenuated when comparing pairs of first cousins and full siblings.

**Conclusions:**

High educational level and income in adulthood, as well as high neighborhood socioeconomic status, may represent protective factors against alcohol use disorders, even when shared familial factors, e.g. childhood socioeconomic status and genetic factors, have been taken into account.

## Introduction

Alcohol consumption is related to a wide range of negative acute and chronic health consequences and causes a considerable part of the global burden of disease [1]. The concept of alcohol use disorders (AUD) includes mental and behavioral disorders caused by alcohol, as well as alcohol-induced somatic complications, e.g. liver diseases, cardiomyopathy and pancreatitis [2]. Globally, alcohol and drug abuse was associated with 6.6% of the disease burden in men in 2015; the corresponding disease burden for women was only 2.0% [3]. Despite the lower absolute disease burden, women seem to be more vulnerable to the harmful effects of alcohol at high levels of consumption [4].

Many studies have found that AUD is more prevalent in disadvantaged socioeconomic groups [5–7] and low childhood SES is associated with later AUD [8]. However, the association is complex and may be affected by sex and levels of alcohol consumption, which has been shown to be higher in affluent and more educated populations [9, 10]. Adolescents with higher parental income and education also have higher rates of binge drinking [11]. Earlier studies have shown that less favorable alcohol consumption is related to social downward mobility [12, 13]. To some extent, the association between low SES and later AUD may be affected by the age of first intake, as initiating alcohol use at an early age increases the likelihood of later AUD [4, 14, 15]. In addition to individual SES, living in a deprived neighborhood may increase the risk for AUD, even though alcohol consumption has been shown to be higher in affluent neighborhoods [16–19]. A review of the literature of alcohol consumption, AUD and SES concluded that the association between these is complex, and that there is a lack of studies exploring this relationship in depth [5]. The present study will make a novel contribution to the field by studying the relationship between different socioeconomic indicators and AUD in both males and females at different ages.

AUD is well known to aggregate in families, through both genetic factors and shared environment in childhood and adolescence, although alcohol habits are also influenced by an individual’s unique environment [2, 20]. One difficulty when studying SES and AUD is to determine whether low SES leads to later AUD, or whether the association is confounded by the action of genetic factors and familial environmental effects, which affect the individual’s future SES and in turn increases the risk for AUD. With the purpose to reduce the effect of familial confounders, we aimed to disentangle the relationship between SES and AUD by using a co-relative design. The advantage of this design is that we were able to take childhood SES into account, by comparing siblings and cousins with an identical or similar shared childhood SES but with differing SES in adulthood. By comparing siblings, we could control for 50% of the genes and their shared rearing environment. The study will also illuminate whether the measured socioeconomic factors may be protective against AUD at certain stages in adulthood. Factors like education, work and civil state may influence an individual’s life style, habits and disease incidence. These factors vary across the life span, and we have therefore measured socioeconomic variables at different ages in adulthood.

The first aim was to study the potential impact of SES, as measured by education, income and neighborhood SES at different ages in adulthood, on AUD, in a large Swedish national sample based on medical, criminal and pharmacy registers, with a mean follow up between ten and fifteen years. The second aim was to explore to what extent the potential associations between SES and AUD are confounded by shared familial factors, e.g. childhood socioeconomic environment and genetic factors, by using a co-relative design.

## Materials and methods

### Measures

We collected information on individuals born 1950–1980 from Swedish population based registers with national coverage. The registers were linked using each person’s unique identification number. To preserve confidentiality, this ID number was replaced by a serial number. We secured ethical approval for this study from the Regional Ethical Review Board of Lund University (No. 2008/409). As the registers are owned by the Swedish authorities and made available to researchers only if certain conditions are fulfilled, we are not allowed to share the data.

#### AUD

AUD was assessed from Jan 1, 1973 to Dec 31, 2013, by using information from the following Swedish medical and mortality registers: the Swedish Mortality Register (from Jan 1, 1973 to Dec 31, 2013), the Hospital Discharge Register (national coverage from Jan 1, 1987, partial coverage 1973–1986), the Outpatient Care Register (from Jan 1, 2001), the Primary Care Registry (different coverage for different counties), the Swedish Prescription Register (from July 1, 2005) and the Crime Register (from Jan 1, 1973). In short, we identified AUD cases using International Classification of Disease (ICD) codes for primary and secondary alcohol-related diagnoses recorded in the registries (S1 Table). Our definition of AUD included diagnoses of alcohol abuse, alcohol dependency and secondary alcohol-related diagnoses such as chronic liver disease and alcoholic cardiomyopathy, as well as repeated crimes related to alcohol abuse. Prescription drug information on disulfiram (Anatomical Therapeutic Chemical (ATC) Classification System N07BB01), acamprosate (N07BB03), and naltrexone (N07BB04) was drawn from the Swedish Prescription Register. Data on crimes with at least two convictions of drunk driving or being drunk in charge of a maritime vessel was obtained from the Crime Register and Suspicion Register. Prior AUD was defined as an AUD registration prior to the age from which we started the follow-up.

#### Education and income

In Sweden, most people complete compulsory school, which is 9 years’ long. From the 1980s and onwards, the majority of people also complete upper secondary school (i.e., two or three additional years). The education variable was primarily based on the number of years of education (1:<9 years; 2: 9 years; 3: 10–11 years; 4: 12 years; 5: 13–15 years; 6: 16 years or more; 7: PhD/ licentiate degree; group 5,6 and 7 include education at university level). In order to be able to compare the variable over time we standardized the variable around the mean (mean 0 and standard deviation [SD] 1) by sex and year.

Information on disposable income was drawn from Statistics Sweden, who has determined a weighted system of income to compare purchasing power parity between different households in relation to the composition of the family, such as number of children. The system is also based on estimates of consumption data. Individualized disposable family income was defined as general family income, including benefits such as child allowance, housing allowance and social assistance, minus current taxes divided by the number of people in the family. In order to be able to compare the variable over time we standardized the variable around the mean (mean 0 and SD 1) by sex and year.

#### Neighborhood deprivation

Neighborhoods (as defined by Statistics Sweden, the Swedish government-owned statistics bureau) are called Small Areas for Market Statistics (SAMS). There are approximately 9,200 SAMS throughout Sweden, with an average population of 1,000. These SAMS units were initially created by the Swedish authorities for administrative as well as marketing purposes. We created a neighborhood social deprivation (neighborhood SES) index for each of the SAMS neighborhoods based on register data for all residents in the neighborhood aged 25–64; this age group was used for the index as they are considered to constitute the working population although students and others who were not working were also included. The neighborhood SES composite contained the following derived at baseline: the proportion of residents with low education (9 years or less), the proportion of residents with low household income (below half the median income), the proportion of unemployed residents, and the proportion of individuals on financial assistance [21]. In the model the composite was kept as a continuous variable, with the SD score ranging between -3 and 11 with higher values indicating greater levels of neighborhood deprivation.

#### Psychiatric diagnoses

Prior psychiatric diagnosis before start of follow up was defined as an ICD diagnosis for any psychiatric disorders except from those related to alcohol and substance use disorders, dementia and mental retardation.

### Study sample and follow up

The dataset included all individuals born in Sweden between Jan 1, 1950 and Dec 31, 1980. No exclusion criteria were applied. For income and education, we had yearly information from 1990 to 2013 while for neighborhood SES we had yearly information from 1986 to 2006. In the dataset, we included income, education and neighborhood SES measured at age 25, 30, 35 and 40 years of age. This created a number of sex- and age-specific strata that each included between 734,227 and 1,209,377 individuals. The strata consisted mainly of the same individuals at different ages, and new individuals were added or removed, to fit the birth cohorts. This means, for example, that information on education for individuals born 1950 was collected at the age of 40 in the year of 1990, and for individuals born in 1980, at the age of 25 in 2005 and at the age of 30 in 2010. The individuals were followed from these ages until they were diagnosed with AUD or until the end of follow-up (death, emigration or the end of 2013). As all Swedish residents have a unique identification number, the follow-up was complete; there were no dropouts or missing data at later time points.

### Statistical methods

We used Cox proportional hazards models to investigate the risk of AUD as a function of income, education and neighborhood SES. In the first model, we investigated the risk of AUD from age 25 until end of follow-up in relation to 1 SD increase in educational level at age 25. In model b, we controlled for previous AUD (defined as an AUD registration prior to the age from which we started the follow-up time). We repeated these models for income and neighborhood SES In model b, we also controlled for individual income and education for the exposure variable neighborhood SES. Finally, in a supplementary analysis, using educational level as outcome, model b was also controlled for prior psychiatric diagnosis.

We replicated these models but investigated income, education and neighborhood SES at age 30, at age 35 and age 40. The follow-up time for AUD was then also changed from age 30, 35 and 40. In the models, we investigated whether the association between income, education and neighborhood SES and AUD varied across time. We therefore allowed the effect of the SES measures to vary based on time. In order to allow us to compare our results, we used the same time-intervals in each model. We allowed one effect during the first 5 years of follow-up, one additional effect during the next ten years of follow-up and finally one additional effect the final years of follow-up. Robust standard errors were used to adjust the 95% confidence intervals (CI) in order to take into account that the sample contained individuals from the same family. We observed minor violations of the proportional assumptions that are illustrated in the Supporting information (S1–S3 Figs).

#### Co-relative analyses

We sought to assess the degree to which the results were confounded by familial risk factors, i.e. genetic factors and/or shared environmental factors such as childhood SES, by using a co-relative design. Using the Swedish Multi-Generation Register, we identified all full-sibling sets and all first-cousin pairs. We used a stratified Cox regression model, in which we refitted all analyses within strata of the defined relative sets (full-sibling sets and cousin pairs). Only sets within the same sex in which the members differed in their exposure to our variable of interest would contribute to the regression estimates. Within each stratum, the hazard ratio (HR) was adjusted for the familial cluster, and, therefore, accounted for an array of unmeasured genetic and environmental factors shared within the relative set. All statistical analyses were performed using SAS 9.4.

### Subanalysis of drinking behavior

As drinking behavior in adolescence may affect the association between SES and AUD, we collected data on drinking behavior at the age of 18 in a subsample of 44,894 men born in 1951. This data was drawn from the Military Conscription Registry, and previous findings based on this data have shown an association with AUD [15]. For these men, we replicated the analyses between AUD and income, education and neighborhood SES at the age of 40 (in 1991), and included drinking behavior at conscription as a covariate in the model. Drinking behavior was assessed as an alcohol score, which was constructed after a factor analysis based on the following 7 questions: “How often do you drink medium/strong beer?”, “How much do you drink when you drink medium/strong beer?”, “How often do you drink wine/strong wine?”, “How much do you drink when you drink wine?”, “How much do you drink when you drink liquor?”, “How often do you drink so that you feel drunk?”, “Do you often get a hangover?” and “Have you ever been arrested for drunkenness?” (S3 Table).

## Results

Table 1 illustrates the number and basic characteristics of individuals in each of the sex- and age-specific strata. P25 M is the general male study population when education was measured at age 25, and P25 F is the corresponding female population. The corresponding study populations containing cousins and siblings within the same sex, with discordant values for the variable of interest, were smaller but still large (e.g. 135,322 male siblings with discordant education). Mean follow-up ranged from 10.5 years to 15.1 years. Both prior (before start of follow up) and total AUD was more common in males than in females at all ages, e.g. 3.7% in the male study population when education was measured at age 25, compared to 1.5% in females. Psychiatric diagnoses were more common in females than in males.

**Table 1:** Basic characteristics of all individuals born in Sweden between 1950 and 1980 divided into sex- and age-specific strata based on the age when socioeconomic status was measured (25, 30, 35 and 40 years old).

	N	AUD (%)	Prior AUD (%)	Mean follow-up, years (SD)	Mean year of birth	Psychiatric diagnose (%)	Siblings*	Cousins**
**Education**								
P25 M	790,654	3.7	1.7	15.0 (6.5)	1972	2.1	135,322/286,459	312,022/624,044
P25 F	746,323	1.5	0.9	15.0 (6.6)	1972	2.8	120,731/254,757	275,924/551,848
P30 M	1,042,989	3.9	2.3	12.5 (7.3)	1969	3.4	192,481/414,040	442,650/885,300
P30 F	985,915	1.7	1.1	12.6 (7.5)	1969	4.5	172,099/368,684	391,473/782,946
P35 M	1,197,586	4.3	2.9	10.7 (8.1)	1966	4.4	226,148/493,732	425,849/851,698
P35 F	1,135,638	1.8	1.2	10.9 (8.3)	1966	5.9	204,622/444,905	380,376/760,752
P40 M	1,205,237	5.0	3.6	10.5 (8.3)	1961	4.9	228,750/507,624	254,433/508,866
P40 F	1,148,159	2.1	1.5	10.7 (8.5)	1961	6.4	209,487/463,025	230,778/461,556
**Income**								
P25 M	794,545	3.7	1.7	15.0 (6.5)	1972	2.2	137,613/291,237	323,677/647,354
P25 F	749,156	1.5	0.9	15.0 (6.6)	1972	2.8	122,900/259,234	284,941/569,882
P30 M	1,047,547	4.0	2.3	12.4 (7.4)	1969	3.4	195,504/420,438	455,711/911,422
P30 F	988,819	1.7	1.1	12.6 (7.5)	1969	4.6	174,696/374,074	401,885/803,770
P35 M	1,202,741	4.3	2.9	10.7 (8.1)	1966	4.4	229,397/500,761	437,335/874,670
P35 F	1,138,528	1.8	1.3	10.9 (8.3)	1966	5.9	207,262/450,422	389,400/778,800
P40 M	1,209,347	5.0	3.6	10.5 (8.3)	1961	4.9	231,666/513,992	261,734/523,438
P40 F	1,150,375	2.1	1.5	10.7 (8.5)	1961	6.4	211,875/468,034	236,296/472,592
**Neighborhood SES**								
P25 M	777,652	3.6	1.6	15.0 (6.4)	1972	2.1	132,924/281,102	311,719/623,438
P25 F	734,227	1.5	0.9	15.1 (6.5)	1972	2.8	118,656/250,115	275,267/550,534
P30 M	890,710	4.2	2.1	13.7 (6.9)	1968	2.9	158,221/339,207	337,748/675,496
P30 F	843,355	1.8	0.9	13.8 (7.0)	1968	3.7	142,404/303,871	300,362/600,724
P35 M	882,446	5.1	2.7	13.4 (7.2)	1963	3.4	158,694/345,449	215,006/430,012
P35 F	840,570	2.2	1.1	13.7 (7.3)	1963	4.4	144,741/313,964	194,316/388,632
P40 M	869,392	6.0	3.5	13.4 (7.3)	1958	4.0	155,713/343,779	85,110/170,220
P40 F	833,149	2.5	1.3	13.7 (7.4)	1958	5.0	143,947/316,882	78,764/157,528

*Number of sibling sets discordant for the SES variable of interest/Total number of siblings

**Number of cousin pairs discordant for the SES variable of interest/Total number of cousins

Education, Income measured in 1990–2012. Neighborhood SES measured in 1986–2006. AUD from 1973 until 2013.

P25 M, male population when education was measured at age 25, P25 F, female population when education was measured at age 25, etc.

AUD, alcohol use disorder

SES, socioeconomic status

At age 25, mean educational level was approximately 12 years (S2 Table). At age 40, the mean educational level was a little lower but it was more common to have a higher level of education. Those who had less than 9 years of education or a PhD/university licentiate degree were relatively few (more than 2 SD from the mean).

### Interpretation of Figs 1–3

**Fig 1:**
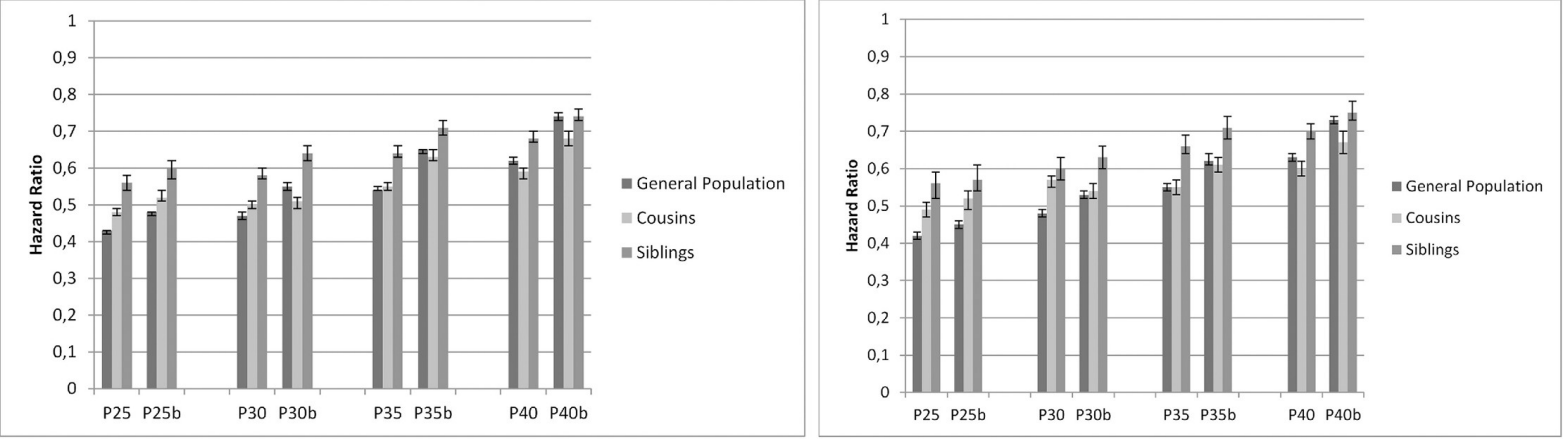
Education. **Cox Regression model with time to alcohol use disorder (AUD) as outcome in relation to educational level at the age of 25, 30, 35 and 40. Vertical axis show HR for 1 SD increase in educational level.** P25, P30, P35, P40 = Population aged 25, 30, 35, 40 years b = Model b adjusted for prior AUD 1a.MALES 1b. FEMALES.

**Fig 2:**
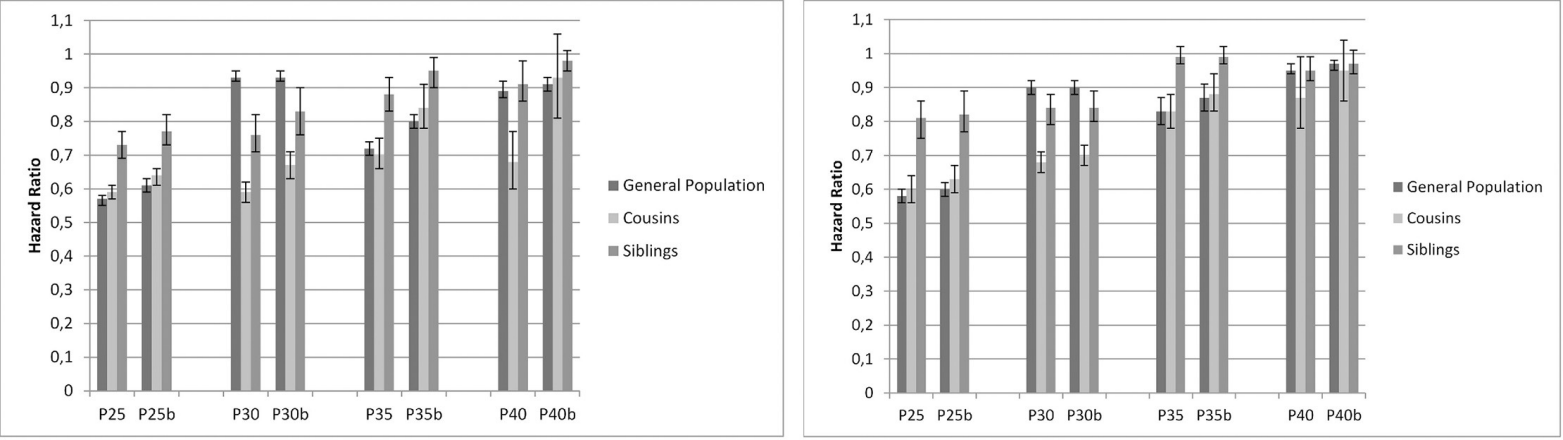
Individualized family income. **Cox Regression model with time to alcohol use disorder (AUD) as outcome, in relation to income at the age of 25, 30, 35 and 40. Vertical axis show HR for 1 SD increase in income.** P25, P30, P35, P40 = Population aged 25, 30, 35, 40 years b = Model b adjusted for prior AUD 2a. MALES 2b. FEMALES.

**Fig 3:**
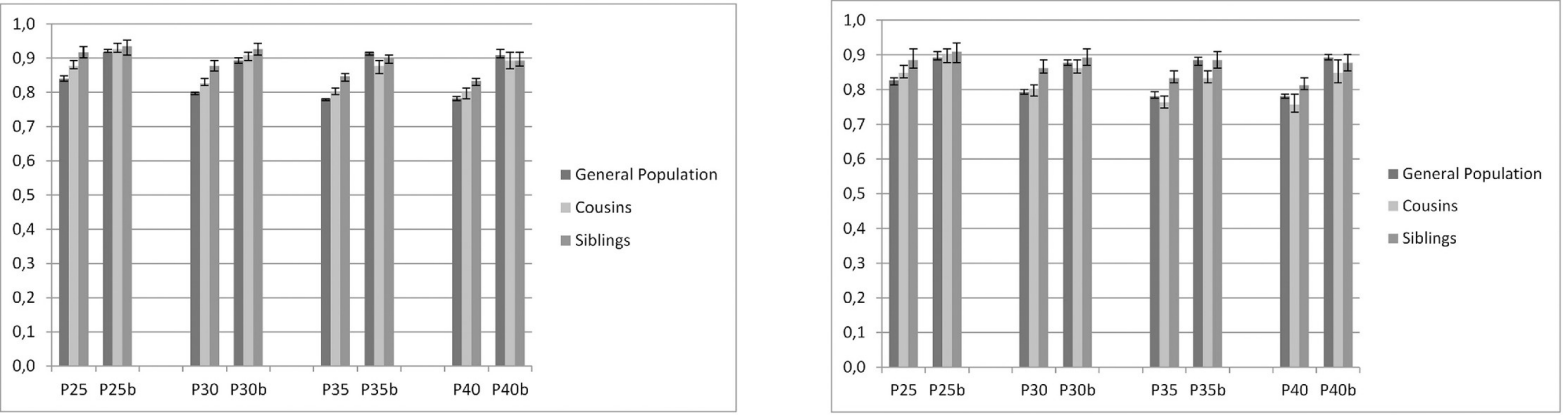
Neighborhood socioeconomic status (SES). **Cox Regression model with time to alcohol use disorder (AUD) as outcome, in relation to neighborhood SES at the age of 25, 30, 35 and 40. Vertical axis show HR for 1 SD decrease in neighborhood SES (continuous variable).** P25, P30, P35, P40 = Population aged 25, 30, 35, 40 years b = Model b adjusted for prior AUD, education and income. 3a. MALES 3b. FEMALES.

Figs 1–3 show the results of the co-relative analyses that examine the potential effect of education, income and neighborhood SES on individual outcomes of AUD. If the association between SES and incidence of AUD is not affected by shared familial factors, e.g. childhood SES and genetic factors, one would expect that the association would be of similar strength in the general population as in relative pairs discordant for their SES. However, if the association between SES and AUD results partly or entirely from familial confounding, the association would decrease substantially in genetically related family members.

### Education

#### General population

As shown in Fig 1A and 1B, higher education was associated with a reduced risk for AUD for both males and females and in all ages. In P25 M, the HR was 0.43 [95% CI: 0.42; 0.43] per SD increase in educational status, and in P25 F, the HR was 0.42 [0.41; 0.43]. In P40 M, the HR was 0.62 [0.61; 0.63] and in P40 F, the HR was 0.63 [0.62; 0.64)]). When controlling for prior AUD, the effects remained but became a little weaker (model b). Adding prior psychiatric disorders to the model did not change the magnitude of the results.

#### Cousins and siblings

Compared to the general population, the HRs for education predicting AUD were greater (that is closer to unity) in cousins and siblings with discordant values of education. The reduction in AUD risk was stronger in the youngest age strata; in male cousins, the HR was 0.48 [0.47; 0.49] per SD increase in educational status, and in male siblings, the HR was 0.56 [0.54; 0.58]). When controlling for prior AUD the risk reduction was attenuated, and, in the oldest age stratum, the HRs among siblings were almost identical to the HRs in the general population. The results indicate that the protective effect of higher education remained but was confounded to some extent by familial factors in higher ages.

#### Follow up time variations

Our test for the proportionality assumption failed to a minor extent, as the association was somewhat weaker (closer to unity) with longer follow-up, but still higher education was associated with a reduced risk for AUD (Supporting Figs).

### Individualized family income

#### General population

Higher individual income was associated with a reduced risk for AUD in both males and females and in all ages (Fig 2A and 2B). However, in the oldest age stratum, the HR was close to unity. The HR for AUD in P25 M was 0.57 (0.55; 0.58) and in P40 M 0.89 (0.87; 0.92).

#### Cousins and siblings

Compared to the general population in P25, the HRs for income associated with AUD were greater (that is closer to unity) in cousins and siblings with discordant values of income. The HR for AUD was 0.59 (0.57; 0.61) in male cousins with 1 SD higher income, and 0.73 (0.69; 0.77) in male siblings with discordant values of income, indicating that the potentially protective effect of higher income at the age of 25 remained also when familial factors had been taken into account.

In contrast to educational status, the pattern in P30 showed a weaker risk reduction of income in the general population than in cousins and siblings. Apart from this, the pattern of income was similar to the one of education. At age 40, the potentially protective effect of income almost disappeared, with HR close to unity in both cousins and siblings (model b). The results suggest some familial confounding for the age 25 group, but in older ages the results show no consistent pattern. If modest familial confounding exist, you would usually find the strongest HR in the general population, with decreasing strength in the HRs in cousins and the least strength in discordant siblings.

### Neighborhood SES

#### General population

Higher neighborhood SES was associated with a reduced risk of AUD in both males and females in all ages (Fig 3A and 3B). In contrast to educational status and income, the reduction in AUD risk was stronger in the oldest age groups. The HR for AUD in P25 M was 0.84 (0.83; 0.85) and in P40 M 0.78 (0.78, 0.79) per SD increase in neighborhood SES.

#### Cousins and siblings

Compared to the general population, the HRs for neighborhood SES predicting AUD were weaker (that is closer to unity) in cousins and siblings with discordant values of neighborhood SES, but the reduction in AUD risk remained, i.e. the potentially protective effect of higher neighborhood SES remained even when familial factors were taken into account. The associations also remained after controlling for prior AUD. However, in P35 M and P40 M and in females (except in age stratum 25) the pattern for neighborhood SES was not consistent after controlling for prior AUD, i.e. there was no strong evidence for familial confounding.

### Subanalysis of drinking behavior

Drinking behavior at the age of 18 was taken into account in the subanalysis of 44,894 men (S4 Table). The potentially protective effects of high educational level, income and neighborhood SES only changed marginally, indicating that adulthood SES is associated with AUD independent of drinking behavior in adolescence.

## Discussion

The results of this Swedish co-relative control study indicate that high education, high income and living in an affluent neighborhood in adulthood, are protective factors against AUD even when childhood SES and other shared familial factors have been taken into account.

To our knowledge, no previous study on risk of AUD has investigated the effect of adulthood SES at different ages, and prior studies have been contradictory [5]. Our results are in accordance with a Finnish twin study, which found that higher educational level was associated with lower levels of alcohol problems in young adulthood [22]. The present study also supports previous studies that have found an association between low individual and neighborhood SES and alcohol-related morbidity and mortality [16, 23–26]. In contrast to education and income, the risk reduction related to neighborhood SES was stronger in the oldest age groups. Neighborhood SES is a complex risk factor that may include other mechanisms not measured in the present study, such as neighborhood crime, social contacts, lifestyle factors and psychosocial stress [27]. These factors may affect people’s alcohol drinking behavior. At age 40 it is, however, difficult to distinguish whether living in an affluent neighborhood is a protective factor against AUD or whether lack of AUD itself contributes to movement to more affluent areas. People may have moved between different neighborhoods several times during adulthood, and their drinking behavior may have changed accordingly. Other factors such as divorce and chronic diseases may also contribute to the relationship between neighborhood and AUD by the age of 40. Most studies of neighborhood characteristics are unable to distinguish between whether the neighborhood itself has led to the development of poor health or whether those individuals who are already affected by a disease or have low SES move to these neighborhoods. However, the use of a co-relative design has partly remedied this in the present study.

### Potential mechanisms

A major problem when studying the association between AUD and SES is to disentangle what comes first. Two scenarios have been suggested: 1) low childhood SES or poor school results predicts problematic drinking behaviors in adulthood, and 2) drinking problems during adolescence/young adulthood predicts lower educational level and lower SES in adulthood [8, 22]. For example, a recent study from Finland showed that alcohol abuse in adolescence/ early adulthood was associated with unemployment, lower income and lower educational level [28]. It is also possible that a set of familial factors predispose to both low SES and risk for AUD with no causal relationship between them. A familial predisposition of problematic alcohol use or parental psychiatric disorders may be underlying factors that influence both AUD and low SES [8]. Probably, some individuals have a greater genetic vulnerability, which can be triggered by environmental stressors [29]. However, in the present study the potential effect of SES on AUD still remained after taking familial factors into account, and after controlling for previous AUD, suggesting that adulthood SES is likely to have an independent effect on risk for AUD. Earlier studies have reported that achieved social position is related more strongly to healthy behaviors that class of origin [13]. Education may contribute to healthy lifestyle habits. A recent study from England concluded that educational attainment was the strongest predictor of consumed volume of alcohol and binge drinking frequency [30]. General intelligence and cognitive abilities may act as mediating factors, as well as individual non-shared environmental factors, such as social contacts and hobbies [22, 29].

The protective effect of education and income was more marked at age 25. Risky alcohol behaviors develop gradually over the years and may subsequently affect the individual’s educational level and income. It is also possible that other mechanisms for the association between SES and AUD are more important later in life, e.g. other health problems or life events such as divorce, loss of family members or unemployment.

There are strong associations between AUD and psychiatric comorbidities, including a wide range of mental disorders such as anxiety, mood disorders, personality disorders and attention deficit hyperactivity disorder [31, 32]. It is more common that people with alcohol dependence are affected by psychiatric disorders than vice versa [32]. Psychiatric illness is also related to low SES [33], and it has been suggested that the association between neighborhood deprivation and heavy drinking is mediated by anxiety and depression, according to the tension-reduction hypothesis [26]. To some extent, we controlled for these effects by accounting for psychiatric disorders in a supplementary analysis. Even if psychiatric disorders would contribute to the association between adulthood SES and AUD, the results of our study show a need to tailor preventive efforts against problematic drinking behavior in subgroups with low SES in adolescence and early adulthood.

Sweden's alcohol policy officially aims to reduce alcohol consumption and reducing the harmful effects of alcohol. A government-owned chain of liquor stores is the only store allowed to sell alcoholic beverages that contain more than 3.5% alcohol by volume, and the age limit to buy beverages in the store is 20 years. At most Swedish restaurants and bars, the legal age to buy alcohol is 18 years. The taxes of alcoholic beverages are relatively high and based on alcohol content. The last decades, several liberalizing alcohol policy changes have been made, e.g. it is now allowed to import a larger amount of alcohol than before, the taxes are relatively lower and the producers are allowed to make advertisements. However, an increased affordability of alcohol (lower price and/or higher income) has only shown weak impacts on alcohol consumption or alcohol-related mortality [34, 35]. For example, a recent study of Finnish and Swedish individuals showed that increased affordability was associated with an increased alcohol-related mortality only in Finnish men with secondary education, but not in women or in other subcategories [34]. In the present study, the protective effect of income was less strong in the general population of males aged 30 as compared to cousins and siblings in the same age group, i.e., the effect of income was stronger when we controlled for shared familial factors. A reason behind this may be that the shared familial factors that contribute to an increased risk of AUD also contribute to higher income in this group. Moreover, it is possible that affordability of alcohol may play a role and that alcohol use is more socially accepted in men with high income in this age, i.e., young, often single men with high-performance job careers, than in men with low income.

### Strengths and limitations

The strengths of the present study include the use of a longitudinal co-relative design including both siblings and cousins, and the possibility to control for prior AUD and psychiatric disorders. Moreover, we had access to a nationwide study population with almost complete data with a long follow-up. The Swedish educational system offers free public schooling of uniform quality. This is a strong setting for the aim of disentangling the relationship between individual SES, familial factors and AUD.

One limitation when studying within-relative pairs is that only pairs that differ in the exposure variable (SES) will contribute to the analyses and we do not know whether these pairs are representative for the population. Moreover, when studying siblings, we only control for 50% of the genes, so there is still genetic influences that we could not control for. Also, a co-relative design can only control for familial factors and not for individual environmental experiences that might predispose both to low SES and to AUD. A pattern with a modest degree of familial confounding is generally showing the strongest HR in the general population, less strong in discordant cousins and least strong HRs in discordant siblings. This pattern was not seen in all age-sex combinations. One contributing factor in the oldest age groups could be the selection of cousins, i.e. only cousins with two parents born 1932 or later were included. Moreover, the confounding effect of genetics could be reduced or excluded by epi-genetic explanations in which even identical twins' genetic expression may become different due to expressing or switching off genes that may be related to AUD-related behaviors. However, this is outside the scope of the present study.

As discussed above, we were not able to fully address the question whether problematic drinking behaviors results in low SES or vice versa. Individuals who start drinking in early teens could bias our results. However, we believe that SES measured in young adulthood is a valid measure for the present aim, and the design with multiple measures of SES over the life-course shows a pattern where SES measured in young adulthood has a greater impact on AUD. Other studies have suggested that adult SES may predict AUD better than childhood SES [8]. Furthermore, part of the socioeconomic inequalities in AUD could probably be explained by other covariables. For example, we did not have access to information about parental alcohol/ drug abuse or criminality [8]. Finally, the present results are applicable in Sweden and may only be generalized to other countries with similar socioeconomic distribution, educational system and alcohol politics.

## Conclusions

Higher individual education, income and neighborhood level socioeconomic status in adulthood seem to be protective against alcohol use disorder in both males and females. The association remained in siblings and cousins, which indicates that adult socioeconomic factors are independently associated with AUD, after familial confounders have been taken into account, and after controlling for prior AUD and psychiatric disorders. Preventive efforts against problematic drinking behaviors should be focused on socioeconomically vulnerable subgroups and preferably in adolescence and young adulthood, before AUD is established.

## Supporting information

S1 Fig Education. Cox Regression model with time to AUD as outcome. Vertical axis shows HR for 1 SD increase in educational level. Follow up time variations (0–5 years, 5–15 years, 15+ years). Model b adjusted for prior AUD.
S1a. MALES
S1b. FEMALES.
(TIFF)

S2 Fig Income. Cox Regression model with time to AUD as outcome. Vertical axis shows HR for 1 SD increase in income. Follow up time variations (0–5 years, 5–15 years, 15+ years). Model b adjusted for prior AUD.
S2a. MALES
S2b. FEMALES.
(TIFF)

S3 Fig Neighborhood socioeconomic status (SES). Cox Regression model with time to AUD as outcome. Vertical axis shows HR for 1 SD increase in neighborhood SES. Follow up time variations (0–5 years, 5–15 years, 15+ years). Model b adjusted for prior AUD, education and income.
S3a. MALES
S3b. FEMALES.
(TIFF)

S1 Table Registries and ICD codes for alcohol use disorders (AUD). (PDF)

S2 Table Years of completed education corresponding to standard deviations of the standardized education variable. (PDF)

S3 Table Drinking behavior at the age of 18, subsample of 44,893 men born in 1951. (PDF)

S4 Table Subsample of 44,894 men born in 1951. Cox Regression models with time to alcohol use disorder (AUD) as outcome in relation to education, income and neighborhood socioeconomic status (SES) at the age of 40. Model 1 age-adjusted; Model 2 adjusted for prior AUD, Model 3 adjusted for prior AUD and drinking behavior at the age of 18.
(PDF)
