# Supplementary figures and images for: Socioeconomic status and alcohol use disorders across the lifespan: A co-relative control study
Source: PLoS One. 2019 Oct 17;14(10):e0224127. doi: 10.1371/journal.pone.0224127 (PMC6797188; doi:10.1371/journal.pone.0224127)

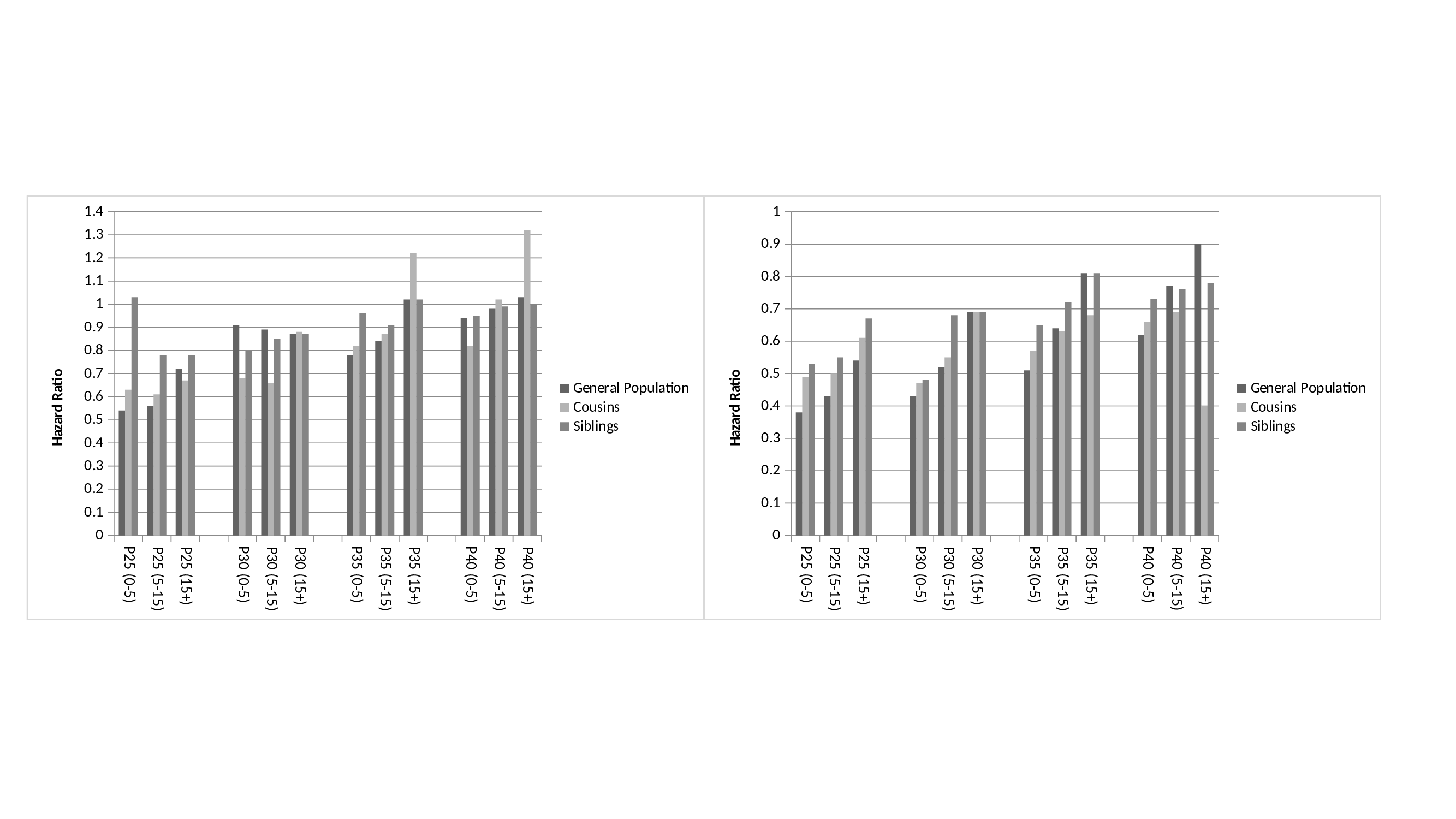

Supplement: S1 Fig — Cox Regression model with time to AUD as outcome. Vertical axis shows HR for 1 SD increase in educational level. Follow up time variations (0–5 years, 5–15 years, 15+ years). Model b adjusted for prior AUD. S1a. MALES S1b. FEMALES. (TIFF) [file pone.0224127.s001.tiff]

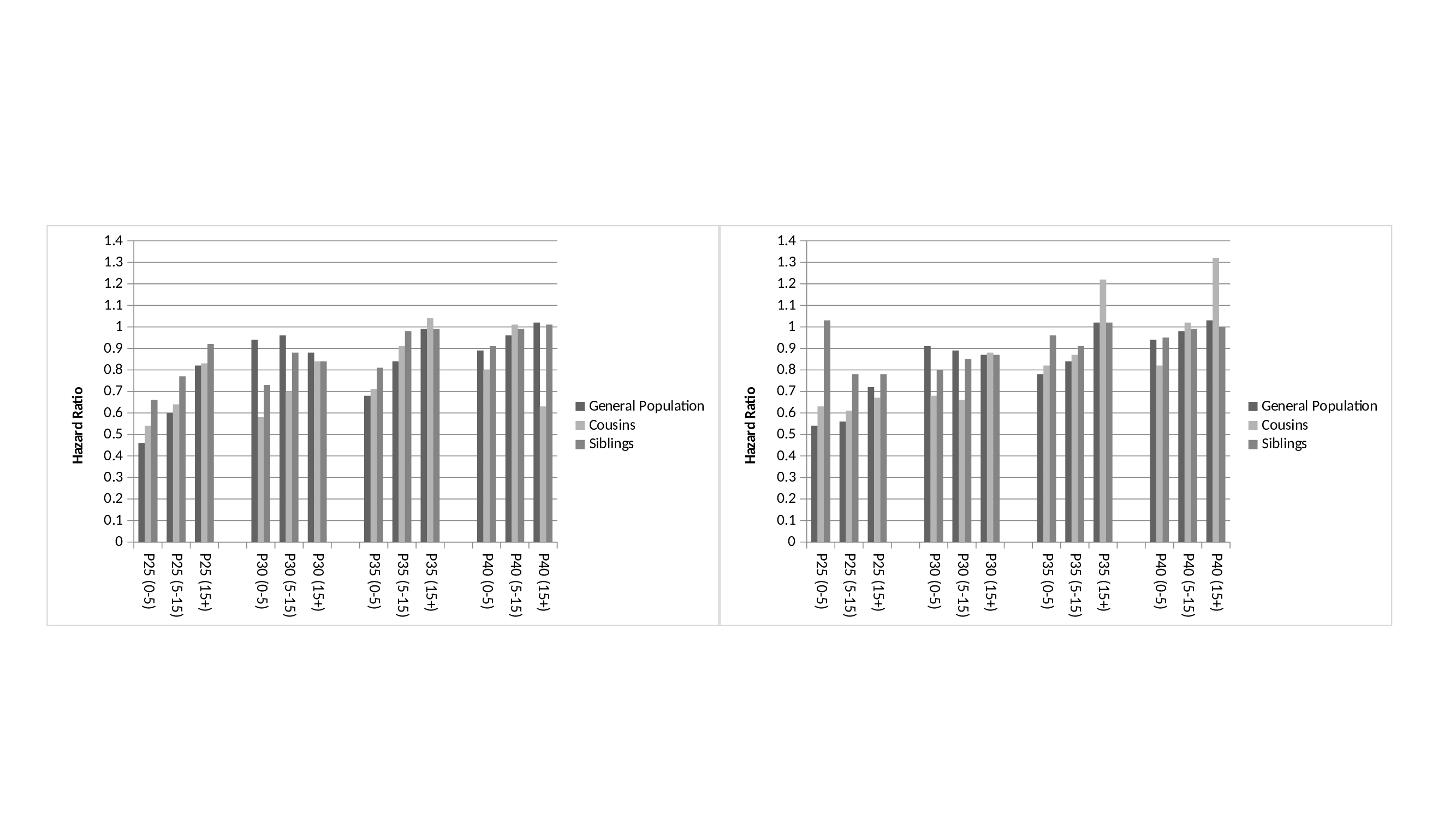

Supplement: S2 Fig — Cox Regression model with time to AUD as outcome. Vertical axis shows HR for 1 SD increase in income. Follow up time variations (0–5 years, 5–15 years, 15+ years). Model b adjusted for prior AUD. S2a. MALES S2b. FEMALES. (TIFF) [file pone.0224127.s002.tiff]

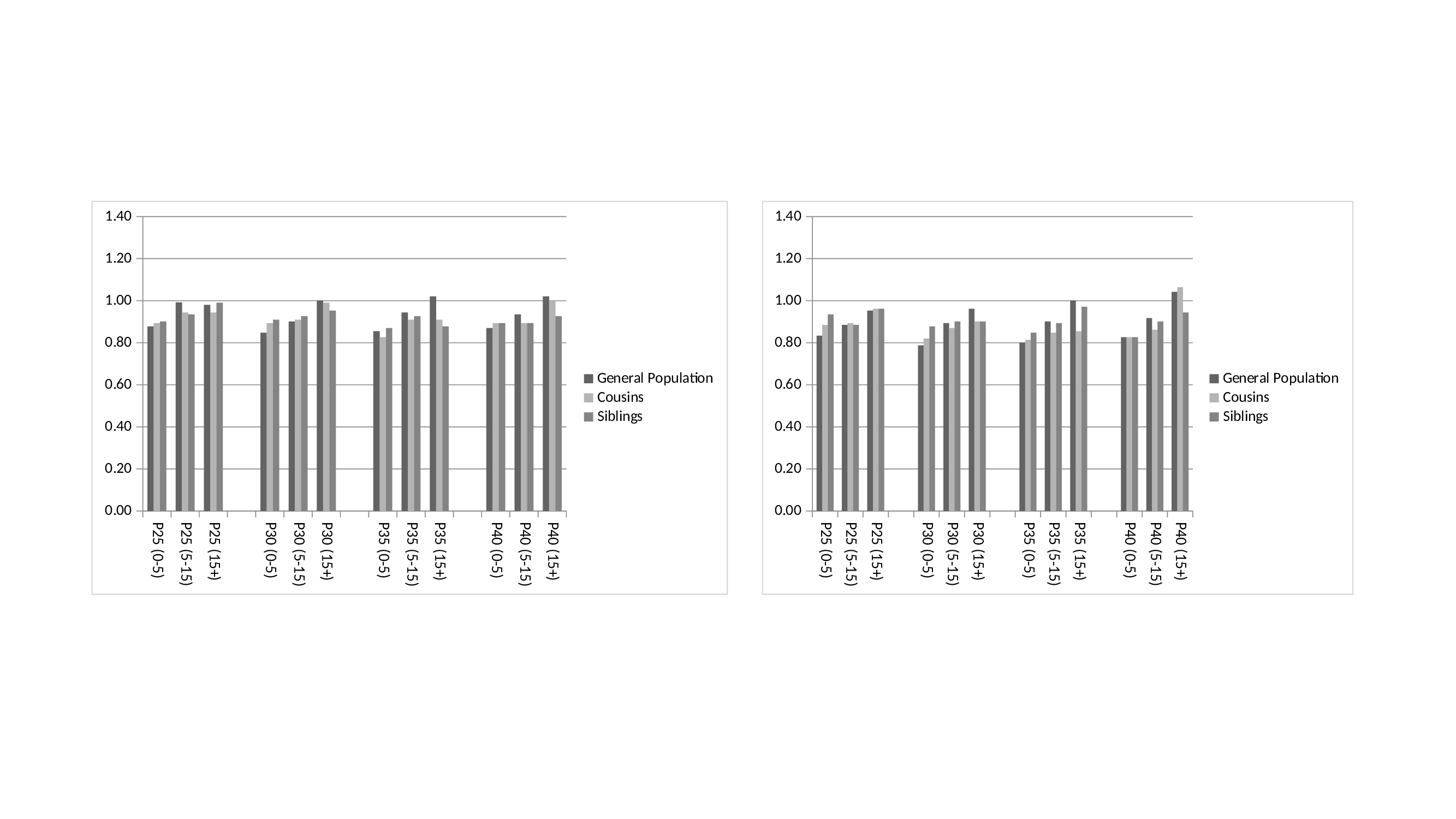

Supplement: S3 Fig — Cox Regression model with time to AUD as outcome. Vertical axis shows HR for 1 SD increase in neighborhood SES. Follow up time variations (0–5 years, 5–15 years, 15+ years). Model b adjusted for prior AUD, education and income. S3a. MALES S3b. FEMALES. (TIFF) [file pone.0224127.s003.tiff]
