# Supplementary material for: Socioeconomic status and alcohol use disorders across the lifespan: A co-relative control study
Source: PLoS One. 2019 Oct 17;14(10):e0224127. doi: 10.1371/journal.pone.0224127 (PMC6797188; doi:10.1371/journal.pone.0224127)
Supplement: S1 Table — (PDF) [file pone.0224127.s004.pdf]

Alcohol use disorder (AUD)	The Swedish Hospital Discharge Register<br>The Outpatient Care Register<br>The Primary Care Registry (PCR)<br>The Swedish mortality Register<br>The Swedish Prescription Register	AUD was identified in the Swedish medical and mortality registries by ICD codes: ICD9: V79B, 305A, 357F, 571A-D, 425F, 535D, 291, 303, 980; ICD 10: E244, G312, G621, G721, I426, K292, K70, K852, K860, O354, T51, F10); in the Crime Register by codes 3005, 3201, which reflect crimes related to alcohol abuse; in the Suspicion Register by codes 0004, 0005 (Only those individuals with at least two alcohol-related crimes or suspicion of crimes from both Crime Register and Suspicion Register were included); in the Prescribed Drug Register by the drugs disulfiram (Anatomical Therapeutic Chemical (ATC) Classification System N07BB01), acamprosate (N07BB03), and naltrexone (N07BB04).
