# Supplementary material for: Socioeconomic status and alcohol use disorders across the lifespan: A co-relative control study
Source: PLoS One. 2019 Oct 17;14(10):e0224127. doi: 10.1371/journal.pone.0224127 (PMC6797188; doi:10.1371/journal.pone.0224127)
Supplement: S2 Table — (PDF) [file pone.0224127.s005.pdf]

Level of eduacation	Age 25	Age 30	Age 35	Age 40
-9 years	-2.44	-2.23	-2.07	-1.76
9 years	-1.62	-1.50	-1.40	-1.28
10-11 years	-0.61	-0.65	-0.65	-0.62
12 years	0.01	-0.14	-0.12	0.05
13-15years	1.04	0.83	0.80	0.83
16 years	1.63	1.42	1.42	1.52
PhD/Lic	2.99	2.30	2.25	2.29
