# Supplementary material for: Socioeconomic status and alcohol use disorders across the lifespan: A co-relative control study
Source: PLoS One. 2019 Oct 17;14(10):e0224127. doi: 10.1371/journal.pone.0224127 (PMC6797188; doi:10.1371/journal.pone.0224127)
Supplement: S3 Table — (PDF) [file pone.0224127.s006.pdf]

Alcohol Score	Factor analysis based on the following variables:<br><br>How often do you drink medium/strong beer? 1=Every day; 2=About once a week; 3=More rarely; 4=Never<br><br>How much do you drink when you drink medium/strong beer? 1=3 cans or more; 2=2 cans; 3=1 can; 4=A couple of glasses; 5=Never drink beer<br><br>How often do you drink wine/strong wine? 1=Every day; 2=About once a week; 3=More rarely; 4=Never<br><br>How much do you drink when you drink wine? 1=1 bottle or more; 2=1/2-1 bottle; 3=1/2 bottle; 4=A couple of glasses; 5=Never drink wine<br><br>How much do you drink when you drink liquor? 1=>35 cl; 2=15-35 cl; 3=5-14 cl; 4=<5 cl; 5=Never drink liquor<br><br>How often do you drink so that you feel drunk? 1=Often; 2=Quite often; 3=Sometimes; 4=Never<br><br>Do you often get a hangover?<br><br>1=Yes, often; 2=Yes, sometimes; 3=No, never If you get a hangover have you ever taken a “pick-me-up”<br><br>1=Yes; 2=No<br><br>Have you ever been arrested for drunkenness? 1=Yes, several times, 2=Yes, sometimes; 3=No never
