# Supplementary material for: Socioeconomic status and alcohol use disorders across the lifespan: A co-relative control study
Source: PLoS One. 2019 Oct 17;14(10):e0224127. doi: 10.1371/journal.pone.0224127 (PMC6797188; doi:10.1371/journal.pone.0224127)
Supplement: S4 Table — Cox Regression models with time to alcohol use disorder (AUD) as outcome in relation to education, income and neighborhood socioeconomic status (SES) at the age of 40. Model 1 age-adjusted; Model 2 adjusted for prior AUD, Model 3 adjusted for prior AUD and drinking behavior at the age of 18. (PDF) [file pone.0224127.s007.pdf]

	**Model 1**	**Model 2**	**Model 3**
**Education**	0.71 (0.69; 0.74)	0.79 (0.77; 0.82)	0.81 (0.78; 0.84)
**Income**	1.00 (0.97; 1.03)	1.02 (0.93; 1.04)	1.01 (0.99; 1.04)
**Neighborhood SES**	0.82 (0.80; 0.84)	0.94 (0.91; 0.97)	0.94; 0.92; 0.97)
